# ‘*I’m not the mother I wanted to be*’: Understanding the increased responsibility, decreased control, and double level of intentionality, experienced by abused mothers

**DOI:** 10.1371/journal.pone.0287749

**Published:** 2023-06-29

**Authors:** Bianca Dekel, Naeemah Abrahams

**Affiliations:** Gender and Health Research Unit, The South African Medical Research Council, Cape Town, South Africa; University of Greenwich, UNITED KINGDOM

## Abstract

A paucity of research has been conducted within South Africa on abused women’s experiences of motherhood, even though abused women tend to be at increased risk of negative physical and mental health difficulties, which can interfere with their ability to take care of themselves and their children. The aim of this qualitative study was to explore women’s experiences of mothering in the context of an abusive relationship. Data was collected via individual, telephonic, semi-structured, in-depth interviews with 16 mothers from three South African provinces, and analyzed according to the principles of grounded theory. Our findings highlight the mothers’ experiences of: a simultaneous increased sense of responsibility with regards to their children and a loss of control over their mothering; as well as experiencing abuse aimed at either the mother or the child, which is simultaneously meant to affect the other; and lastly, mothers’ assessing themselves negatively through normative paradigms of ‘good mothering’, regardless that they often mother in the best way they know how to, given challenging circumstances. Therefore, this study highlights that the motherhood institution continues to create ‘good mothering’ benchmarks against which women themselves evaluate their mothering, often leading to feelings of inadequacy. Our findings also emphasize that the environment created by men’s abuse is in conflict with the great expectations placed upon mothers in abusive relationships. Thus, mothers may experience huge pressure, which may lead to feelings of failure, self-blame, and guilt. This study has demonstrated that the abuse mothers’ encounter adversely impacted on their mothering. We therefore emphasize the need to better understand how mothering is influenced by and responsive to violence. This is important as understanding abused women’s experiences can assist us to further develop appropriate support mechanisms needed to ensure minimal impact on both women and their children.

## Introduction

Worldwide, the burden of Intimate Partner Violence (IPV) remains alarmingly high. Globally, the World Health Organization (WHO) estimated that 30% of women aged 15 and older, have experienced physical and /or sexual IPV at least once in their life, with the Sub-Saharan African region reporting amongst the highest rates [1]. IPV is a challenging and solemn reality for many women in South Africa. A household survey conducted within the Gauteng Province of South Africa, noted that 42% of women reported ever experiencing IPV [2] and 56% of men reported ever having perpetrated physical abuse against a female partner in their lifetime [3]. Global reports on the potential increased risk for IPV during Covid 19 conditions have been reported but not all reports showed increased risk [4]. Locally, the public health measures implemented by the South African Government to curb the spread of the COVID-19 virus, such as stay at home orders and travel restrictions, may have placed women at increased risk of abuse. Specifically, lockdown likely magnified the risk for escalation of abuse in families already experiencing IPV prior to COVID-19 [5]. However, robust research has not been done.

Among the highest levels of IPV are reported by women of reproductive age, shown from studies globally and in South Africa [6, 7]. Therefore, many women need to grapple simultaneously with an abusive partner and with their role as mothers, which is very difficult. For example, compared with women who are not in IPV relationships, abused women are likely to suffer more from mental health challenges, such as anxiety, depression, low self-esteem, and posttraumatic stress, as well as physical injuries, economic hardship, and social isolation [8, 9]. The huge health and economic burden, minimizes women’s resources to self-care and care for others, making it more difficult to perform the already difficult work involved in mothering [10]. Therefore, this type of research is important as the burden as well as the impact of IPV on mothering and the accompanying challenges facing these women, has received much less attention, especially within undeveloped setting [10–15].

The available research on IPV and mothering has found that abusive men may target women’s mothering, in numerous ways, as part of the abuse they perpetrate. Examples of this type of abuse include, but are not limited to, inciting the children against her; belittling/humiliating her in front of the children and undermining her maternal functioning /skills; making threats to report her to Child Protective Services and have her children removed; abusing or otherwise placing her children in danger as a means to intimidate, harm, or punish her; restricting or withholding resources so she cannot meet her children’s needs; overruling her decisions; communicating jealousy of the attention she gives her children; preventing her from comforting and/or caring for her children; and abusing her in front of her children [16–19], thereby undermining her value as a person, her identity and role as a mother, her relationship with her children, and her ability to nurture and protect her children [11]. For many women, violence that threatens the mother role and mother-child relationship may be especially damaging [11].

For instance, the targeting of women’s’ mothering as part of the abuse, may have adverse effects on their functioning as a mother, and as a result, they may display poorer parenting [20], including the abuse of their children [21]. Moreover, since women in most societies remain primarily responsible for the caretaking of children and the household, the targeting of their mothering role as part of the abuse, may exacerbate the stress already associated with the role of mother [22, 23]. Despite the strides made by feminist thought and practice, the majority of home and childcare responsibility remains the domain of women [10]. Therefore, it is crucial to locate the challenges women face in these situations, within an understanding of the social organization of mothering and of the expectations placed on women as mothers. This is important as these factors have an effect on women’s mental health, identities, and the circumstances in which they perform their mothering [24].

We may, therefore, surmise that for many women in abusive relationships, the abuse may increase or heighten their sense of mothering responsibility in diverse ways [10, 15, 25]. For example, some mothers may also have to deal with their children mirroring their partner’s abusive behaviors or need to protect their children from witnessing abuse or being abused [26]; and/or have their challenging situation further exacerbated by their male partners’ shortcomings as fathers [10]. In line with this, research has found that such fathers tend to be inflexible and authoritative [16], uninvolved in their children’s lives [27], self-absorbed and possessive [28], manipulative [16], and physically punitive and unaffectionate [29].

Therefore, for many women in abusive relationships, their situations may make it challenging for them to realize their maternal potential, as there may be a disconnect between their own expectations of motherhood and the reality of the mothering experience, which may lead to internal conflict and numerous adverse feelings such as frustration, guilt, and inadequacy [10]. The few studies found, have emerged from developed settings, and little is known about developing settings including South African women’s experiences. In this paper we address the gaps and present an analysis from a qualitative study that explored South African women’s experiences of mothering within the context of IPV.

## Methods

A sample of 16 women (see Table 1) were recruited using purposive sampling. These 16 women were residents from five shelters, based within both rural and urban areas within three South African Provinces, namely the Western Cape, Gauteng, and Kwa-Zulu Natal Province. These shelters provide residential shelter to abused women and their children, along with supportive counselling, and skills training.

**Table 1:** Participant characteristics.

Participant name	Participant age	Race	Region	Number of children	Children’s ages	Son or daughter	Married or dating	Was the male partner the children’s biological father?	Number of overlapping years of being in an abusive relationship and a mother
Tony	21	Black African	Gauteng	One	8 months old	Son	Dating	Yes	8 months
Caroline	44	Colored	Western Cape	Two	18 years old and 20 years old	Two daughters	Married	Yes	20 years
Ursula	20	Black African	Kwa-Zulu Natal	One	3 years old	One boy	Dating	Yes	3 years
Belinda	44	Colored	Gauteng	Two	24 years old and 20 years old	Two daughters	Married	Yes	24 years
Michelle	46	White	Western Cape	Three	19 years old, 14 years old, and 9 years old	One son and two daughters	Married	Yes	19 years
Marsha	39	Colored	Gauteng	Five	23 years old, 18 years old, 15 years old, 10 years old and 5 years old	Son, daughter, son, son, daughter	Married	Yes	23 years
Lauren	33	Colored	Western Cape	Two	9 years old, 6 years old.	One daughter and one son	Dating	Yes	9 years
Angel	52	Colored	Western Cape	One	21 years old	Daughter	Married	Yes	21 years
Sally	36	White	Kwa-Zulu Natal	Three	10 years old, 3 years old, and 2 years old	Son, son, daughter	Married	Yes	10 years
Elizabeth	30	Colored	Gauteng	Two	7 years and 14 years old	Son	Married	Biological father to the 7 year old. Not to the 14 year old.	7 years
Megan	24	Colored	Kwa-Zulu Natal	Three	6 years old, 5 years old and 2 years old	Two sons and one daughter	Dating	Yes	6 years
Lucy	34	Colored	Gauteng	Four	17 years old, 15 years old, 11 years old, and 5 years old	Two boys and two girls	Dating	No	5 years
Anna	34	White	Kwa-Zulu Natal	One	14 years old	One daughter	Dating	Yes	14 years
Lana	25	Black African	Western Cape	Two	5 years old and 2 months old	Daughter and son	Dating	Yes	5 years
Unity	41	Coloured	Western Cape	Five	21 years old, 19 years old, 16 years old, 10 years old, and 8 years old.	Four sons and one daughter	Married	Yes	21 years
Ingrid	32	Black African	Gauteng	One	4 months old	Son	Dating	Yes	1 year

Ethical approval was obtained from the South African Medical Research Council’s Human Research Ethics Committee and access approval to conduct research within each shelter was provided by each shelters’ management. Thereafter participant recruitment occurred through each shelter’s social worker, who spoke to each woman who met the inclusion criteria (i.e., Women (over the age of 18 at the time of interviews); who experienced IPV from a male partner (who could either be the participants’ former or current boyfriend/husband) and who had at least one child (or more), living in the same home as the woman and abusive male partner); to determine whether she would be willing to participate. If she agreed, a suitable date and time would be organized for the first author to do a more detailed introduction of the study, including consent procedures etcetera., and thereafter to conduct the interview. Written informed consent procedures were followed and the aim of the study as well as all ethical procedures (risks and benefits, anonymity, confidentiality) and the recording of the interviews were explained to participants. To maintain anonymity, we used pseudonyms in the paper.

The first author conducted individual, telephonic interviews, due to COVID-19 restrictions.

These semi-structured in-depth interviews were conducted in June 2020, and was on average, one hour in length. Repeat interviews were done as two interviews per participant were conducted, based on a scope of enquiry, which was developed based on the literature reviewed, and was used to guide the interviews. This allowed the agenda to be flexible although partially directed by the interview schedule. The first interview briefly explored the women’s’ background information and then moved into the start of their intimate relationships and the beginning of their mothering journey. The second interview delved deeper into their relationships with partners and children, and also explored the eventual leaving of the abusive relationship.

We recognized that support for participants was vital. Prior to each interview we asked each social worker whether he/she would be willing to provide support after the interview if needed. However, no participants requested additional support. Psychological support was also arranged for the first author, who conducted the interviews. After the interviews were conducted, the first author transcribed some of the interviews and a transcriber assisted with the remaining interviews. All interviews were transcribed verbatim. Quality checks were done by the first author who conducted crosschecking of all the recorded interviews against the transcribed interviews.

Data analysis was performed according to the principles of grounded theory. During manual open coding, audio-recordings and interview transcripts were reviewed with the aim of examining the text for thoughts, ideas, and meaning and consequently assigning them codes, and consequently a codebook was developed. Emphasis was placed on allowing concepts to emerge naturally without forcing them into predefined categories. Categories were then divided into subcategories. Trimming down of the codes was done with the assistance of the second author, thereby concluding the first stage with 21 codes. Together, a single category was identified (axial coding) as the central phenomenon of interest. This selection was made based on the categories most extensively discussed by participants (i.e., the codes with the highest frequency), which were then positioned as central features, around which, other categories were related, and a storyline was constructed. To illustrate this storyline, discriminate sampling was used, which refers to selecting certain participant quotes, which are able to maximize opportunities for verifying the storyline. The storyline was further validated by searching for relevant literature pertaining to categories. Member checking was performed with participants to iron out ideas and reach consensus. Lastly, core categories were then organized into the research paper. Quotes used herein are illustrative of the codes selected.

### Findings

This study included 16 mothers, whose ages ranged between 20 and 52 years old at the time of the interviews. The women had on average, two children each; and 8 women were married, and 8 women were dating their male partners. Despite women coming from different racial groups, they had similar demographic profiles, living in communities where socio economic hardships were common. Women described their communities as plagued by violence and crime, poor policing, gangs, unemployment, alcoholism, drug addiction, and IPV.

### Mothers’ experiences of enduring various types of abuse and how it impacted on their ability to mother

For most women, the extreme physical violence they experienced, and for some, near femicides, began during their pregnancies. Ingrid (who has a 4 month old son and who was in a dating relationship with her abuser) provided an example of the abuse she endured during her pregnancy, ‘*He* (boyfriend) *would beat me and then make me take my clothes off and kick me out*, *then I sleep on the mat where you wipe your feet before coming in*’. Pregnancies were identified as noteworthy moments in their histories of violence as it often signified the beginning of the abuse.

For most women, the abuse did not end after the birth of their babies and instead, the abuse continued for years and/or months, and often escalated in severity. Thus, the women endured prolonged physical abuse (refer to Table 1 for additional information), which was often described as harsh and sadistic, as evidenced in Caroline’s narrative, who was married to her abuser and who has two daughters, *‘He* (husband) *threw petrol over me and stood over me and he was telling me he is going to burn me to death*. *He kept flipping the lighter on and off*. *I just froze and I couldn’t move because I was so scared’*.

Some of the women also experienced sexual abuse perpetrated by intimate male partners, as explained by Angel, a married, mother of a 21 year old daughter, ‘*He* (husband) *would never take no for an answer’* and *‘When I would be asleep*, *he* (husband) *would have sex with me*’. Compounding this trauma, was that ‘*the sexual abuse would happen right in front of her* (daughter). *As a mother it broke me; that she had to witness that abuse and it was very traumatizing for her*’. The women’s’ narratives were filled with examples of adverse physical health following the abuse, which, coupled with the emotional trauma, often hampered their ability to mother, as Caroline explained, “*After the one time he beat me*, *I was so devastated*. *I couldn’t even breast feed my baby because I was so upset and sore*”. Likewise, Tony explained that she would often, “*need to bath my baby but I’d be too sore to bath him”*.

Another type of abuse experienced was financial abuse; a tactic to maintain control by forcing financial dependency (‘*I was forced to resign by him* (husband)’(Sally)) and, therefore, making providing for children (‘*I really loved my job because I could buy the children stuff*’ (Sally)) as well as the option of leaving the relationship, ‘*incredibly difficult*’.

Participants’ narratives depicted various behaviours that their male partners had engaged in, in an attempt to dominate and control them, showing that their mothering was often used as a target in the male partners’ violence. Most women received near daily verbal thrashings attacking their motherhood, where they were criticized and accused of being ‘bad’ mothers, as Megan (a 24 year old mother of three children) explained, “*He’d tell me I’m a crap mother*, *that someone else should be looking after my children*, *that I don’t know what I’m doing*. *He would undermine me and say this in front of my children”*.

It was not long before the women started believing these insults and accepting them as truths, “*I started believing what he was saying about me… You can’t help but believe it if you hear it every day*” (Marsha, a 39 year old mother of five children). Their narratives provide insight into the psychologically abusive, verbal tactics their partners utilized to discredit them as mothers. Eventually, the abuse and internalization thereof, began adversely affecting their parenting as several women mentioned that the abuse prevented them from caring for their children in a way they had wanted to. Belinda (44 year old mother of two children) explained, “*He would tell me that I’m a bad mother*. *Eventually*, *deep down*, *you start believing it*, *then you start mothering from a guilty point of view*. *So now it’s like I believe I’m a bad mother and because I’m guilty of being a bad mother*, *my child starts getting treated differently*, *so whatever she asks for*, *I make sure she gets it*, *even if it isn’t in her best interest*”. It is not uncommon, in homes affected by IPV, for mothers to face difficulties in establishing limits or setting boundaries for their children.

### The double level of intentionality

Participants sobbed while expressing the emotional trauma they endured when their male partners abused their children, which they knew was part of a strategy to emotionally and psychologically harm them. Sally explained, ‘*There was often times where it wasn’t necessary to hit them and*, *in these times*, *he would be smiling*, *and he told me he knows it breaks my heart*. *Like pure evil hey*? *I know he was doing it to get to me and it worked*’.

The women also elaborated on the difficulty and stress involved in trying to remain silent when one’s children are being beaten, as evidenced by Unity (who was married to her abuser and is a mother of five children), ‘*When I saw how he was beating the children*, *the things that I feel and want to say*, *I can’t even explain*. *Like if I see him beating them with the belt*, *it feels like he is beating my body*. *One of the hardest parts was keeping my mouth shut’*. Similarly, it appears that some children had a comparable experience when seeing their mothers beaten, as Anna (who was in a dating relationship with her abuser and has a daughter) explained, *‘My daughter told her father*: *‘what you are doing to my mother*, *you are also doing to me’*. *That was shocking to me*. *She meant like the pain I am feeling*, *she is also feeling*, *like emotionally’*. Indeed, Megan, who was also in a dating relationship and is a mother of three children, elaborated on the abuse she experienced, which was often intended to upset her children, ‘*He* (boyfriend) *used to abuse me in front of them*. *He used to like it if they cried*. *Then he would feel like his job is done*. *He has now abused me and made them cry*’.

In addition, the women recounted many stories about how their abusers emotionally abused the children by blaming them and their behavior as the reason for his use of violence against their mothers. For example, Belinda said that, ‘*If my daughters upset him*, *he would take it out on me… … and I remember him shouting*, *‘this is your fault*, *now your mommy must suffer’*. *Then he hit me in front of the children*. *He wanted them to see*. *The children were screaming for him to stop*. *He told them*, *‘Next time you’ll listen hey*?*’ so he actually hit me to make them upset*’. Indeed, it is not uncommon for abusive men to externalize blame to excuse themselves from taking responsibility for their violence and other tactics of coercive control.

Most women reiterated Unity’s thoughts as to how and why the double level of intentionality occurs, ‘*He would beat the children for two reasons*. *One*, *because he knows it is going to pain me*. *Two*, *because he hopes I intervene*, *and if I do*, *then he can beat me*. *So*, *he would beat them because he knows my weakness*, *my children*, *so beating them for unnecessary things just for me to intervene then he can start with me because he never had a real reason of fighting with me because I always did my best to be a good mother and partner’*.

The following excerpt from Sally, also elucidates the double level of intentionality as well as the lack of empathy and concern for the women’s mental health, displayed by the men, ‘*It depends on me*, *like if I want him to be nice to my children*, *then I must be nice to him*. *I must make sure he’s happy*, *like have sex with him and make sure his mouth is moving* (provide food), *so that his relationship with them is okay*. *Their relationship is like dependent on how I behave towards him’*. She further explained that the children will endure abuse if she upset him.

### Increased sense of responsibility for children, yet loss of control over mothering

Most of the women’s experiences highlighted their partners’ overall lack of interest in the children, beginning with their pregnancies and continuing once their children were born. For instance, many women explained that their male partners rejected paternity, which may have been a tactic to hurt the women, saying, ‘*‘this is not my baby’* (Tony, who was dating her abuser at the time and has a 8 month old son); and many of their partners were not interested in partaking in the naming of their children, ‘*He refused to give the child a name*. *I was very disappointed because I thought as a father*, *he would want to give his son a name’* (Ingrid); which reiterated their possible lack of accountability.

In addition, most participants mentioned that they had for the most part, not been able to rely on their partners to help them perform the work involved in parenting. This often began when the women were pregnant, and conjured feelings of ‘*loneliness*’ because ‘*he wasn’t there for me*. *He would never accompany me to the clinic for check-ups*. *Even when I gave birth*, *he didn’t come to visit*. *Then once we got home it continued*, *he never even held the baby’* (Ursula, who was in a dating relationship and has a son). This lack of paternal support (which one could argue bordered into selfishness/self-absorption) and maternal responsibility dynamic, largely extended after the children were born, as evidenced by Marsha, ‘*He would even go as far as eating the children’s last porridge*, *then there was nothing for them*, *then I had to go out and beg for anything*. *Because I knew before I left in the morning*, *there would be food for them*, *but when I got back*, *he has eaten everything… Then the stress and responsibility to find something for them to eat falls on me*’. Indeed, this increased responsibility was summed up well by Lauren, who was dating her abuser and who has one daughter and one son, ‘*I had so much on my plate*. *I had to try for me and my children not to be abused*, *I had to tend to my children*, *I had to cook and clean*, *make sure he is happy*, *and I must think what I can and can’t say all the time*’.

Although the women had an increased sense of responsibility, they also experienced a simultaneous loss of control over their mothering. Almost all the women explained that their male partners showed minimal interest in the children (‘*did nothing for my baby*’ (Ingrid)), yet made the rules and decisions (‘*Once my baby was born*, *he made all the rules’* (Ingrid)), which one may argue was a way to enforce control and domination. Participants also emphasized the accompanying feeling of frustration and loss of control, as Megan explained, ‘*He* (boyfriend) *would teach them* (children) *if someone hits them*, *they must hit back*. *I would tell them not to do that*. *The worst part is that the children would listen to him*, *and I am not seen*. *Like what the father says is right*. *I felt like a nobody*, *like I had no control over how our children was raised*’.

Some male partners also used more subtle manipulation strategies to challenge the mothers’ authority and to further remove her sense of control. For example, the women were often stripped of their ability to discipline, as Megan explained, ‘*If my children were naughty*, *he would say he is the only one that can punish them*. *I am a nothing*, *he doesn’t even see me as their mother*. *But I must bath them*, *feed them*, *clothe them*’. At the same time, the women felt that they needed to comply and ‘*agree to keep the peace*, *but not really agreeing deep down inside*’ (Sally) in the hope of preventing abuse. This was a consistent theme within their narratives: these mothers were in a daily process of protection centered on creating a violence free environment, which meant monitoring both their children but also more widely, the general circumstances that may precipitate violence.

Indeed, Belinda stated that, ‘*Everything was his rules for the kids*, *but I had to enforce them and ensure the kids respect it*, *otherwise he will kill me*’. Within the women’s narratives, it appears that these men were the controllers, the one’s making the rules, and the women had to enforce these rules or else abuse was likely to occur.

The men also simultaneously worked hard at breaking their female partner’s confidence. Ingrid explained that her partner often told her, *‘You know nothing’* about being a mother and said, ‘*It hurts so much and made me lose confidence in being a mother*’. The various types of abuse that these women endured, appeared to undermine their maternal and self-confidence and tended to exacerbate their already impoverished view of themselves as women and as mothers.

### Inability to protect children as well as themselves from abuse and accompanying negative feelings

The mothers explained the frustration they felt in not being able to protect their children. As Unity explained, ‘When he beats the kids, that’s when I feel at my lowest, because I cannot protect them, I cannot even protect myself. When he is beating the kids… I must run because I’m next. Then he knows he got what he wanted because I am in pain when he is beating my children’. Tied to this, many of the women elaborated on the accompanying feelings of guilt, as outlined by Michelle, ‘I’m not the mother I wanted to be because I was not able to protect my children from being abused or from seeing me be abused. I will always sit with that guilt and feeling like I was never good enough. I was always failing because we were both always abused’.

Many women also reiterated Unity’s sentiments in that they felt as though they had to meet certain expectations to be ‘*good enough*’ mothers and partners and *‘did everything he asked’* in an attempt to protect their children and themselves from being abused. Part of the emotional trauma these women faced was feeling as though they had to constantly be alert and vigilant and in a ‘*protective mode*, *trying to always protect’* (Angel) their children. A common reason for this was also feeling as though they never truly *‘knew what he* (husband) *was capable of’* (Sally) and had concerns about *‘him murdering my children’* (Sally) or *‘raping my daughter’* (Belinda) and thereafter *‘blaming the drugs’* (Sally and Belinda). Within the women’s’ narratives, it becomes evident that their male partners lack of interest and responsibility, was compounded by their misuse of alcohol and drugs, which the women viewed as increasing the risk of violence towards their children. Therefore, not only were these mothers unable to rely on their partners to assist and support them in parenting, but these men were also potentially dangerous for their children to be around.

In addition to trying to prevent their children from being exposed to violence and/or from being abused, was the simultaneous need to respond to their children’s emotional needs by helping them to make sense of the situation. However, the mothers explained that just as they were unable to physically protect their children, they were also mentally unable to respond to their children’s emotional requirements. Lana, a mother of two and who was dating her abuser, explained that, ‘*My daughter* (aged 5) *would cry and scream when she sees him abuse me*. *Then she would ask like*, *‘why did he do that*?*’*. *Then*, *I don’t know how to answer*, *to explain it to her*, *like I don’t have the energy*. *That made things worse and reminded me how I failed as a mother*’. Similarly, Belinda noted that, ‘*I didn’t conversate with my kids*. *I avoided my children because there’s no way I was going to open up myself to really hear what my child is feeling*. *I couldn’t bear questions*. *It was too emotionally difficult*. *Yoh it made me feel like I wasn’t good enough*’. It is also not uncommon for women in abusive situations to prioritize achieving physical safety for children over meeting emotional needs.

Overall, the women explained that they felt as though they could never meet the expectations placed upon them, which contributed to a sense of feeling inadequate as both a mother as well as a partner, as evidenced by Tony, ‘*I had so much responsibility on my shoulders hey*, *but that man gave me no confidence to do anything*. *He just broke me every day*. *I always felt like I wasn’t good enough as a girlfriend or a mother*’. Their stories echoed their sense of self-criticism as evaluated as deficient as partners and mothers, entrenched in, and engendered by, narratives of ‘good mothering’. Their accounts reflect their challenges and experiences of guilt and inadequacy, yet their continuous dedication to mother, reflects the difficult predicaments inherent in the intersection between the ‘good mother’ idea and the complexity of mothering while being in an abusive relationship.

### Children mirroring mothers’ emotional experiences and/or their partner’s abusive behavior

Just as the mothers attempted to protect their children, some children also attempted to protect their mothers, albeit through the use of violence. Elizabeth (who was married to her abuser), in referring to her 7 year old son, said, ‘*I woke up with my husband lying next to me and he wanted to have sex with me and I refused so he got up and took out a knife*. *At that moment my son woke up and told his father*, *‘Tonight I will murder you*, *tonight I am going to commit a murder’*. *I felt like somebody was protecting me’*. Likewise, Lauren (who was dating her abuser) explained that, ‘*We got into an argument and then my daughter* (age 7 at the time), *stepped between us*, *she said she won’t allow him to hit me*, *and then he pushed her away and she fell*, *and he wanted to come for me*, *and then she got up and took a knife and stabbed him*’. Elizabeth and Lauren’s excerpts allude to the notion that mother–child relationships are not unidirectional. Indeed, numerous participants mentioned that the abuse they experienced had impacted on their children’s attitude and behaviours, which had, in turn, affected their experiences of mothering. This was significant when the mothers felt that their children had either firstly, reproduced their male partners’ abusive behaviours or secondly, copied their own behaviour.

For example, some male children started mimicking the aggressive behaviour (amongst friends and siblings) displayed by their fathers or mothers’ intimate partners. When referring to her 7 year old son, Elizabeth said that he ‘*was becoming very abusive towards the other children*’ (Elizabeth). Likewise, Megan explained that, ‘*Both my sons* (aged 6 and 5) *use to hit their sister* (aged 2). *Copying their father…*. *They see what their father is doing to me*, *so they feel they can hit their sister*. *It made me feel like I failed as a mother*’. Sally also referred to her children’s mimicking behaviour and said it, *‘made me so sad*, *made me feel like I failed…*. *and felt guilty like I was never a good enough mother because if I was*, *they would never do that copycat stuff*’.

In line with this, mothers also worried that their sons would grow up to abuse their wives and that their daughters would be victims of IPV. Megan explained, ‘*What is concerning to me is that if my son at 6 years old has adopted aggressive behavior*, *one day he must also take a wife and raise children*. *I am concerned that it may affect him when he is older*, *and he’s already fighting with boys and even hitting girls*’ (Megan). Lauren also mentioned that, *‘With children it’s like ‘monkey see monkey do’*. Indeed, the mothers were worried about the consequences of their children’s exposure to and often experience with violence, which is understandable given the positive association between adverse childhood experiences such as, witnessing domestic violence, and IPV perpetration in adulthood [30].

Secondly, some daughters also began displaying similar behaviour to their mothers’. For example, according to Anna, ‘*My daughter* (aged 14) *became a lot like me*. *She was withdrawn and kept to herself*’. Likewise, Caroline stated, in referring to her then younger daughters, that they, ‘*was like me*. *They started crying a lot and not talking … just keep everything inside*’ indicating similar emotional impacts from the abuse.

## Discussion

This paper explores the complex challenges that abused women experience in trying to perform the already difficult task of mothering. This research builds on our understanding of the manner in which a male partner’s violence adversely influences a woman’s mothering and her relationship with her children. Few in-depth studies have been conducted outside of developed settings to explore these issues and therefore this research is particularly valuable within the South African context, given the high levels of IPV [2, 3] and child abuse [31].

In order to develop a deeper understanding of the difficulties abused women face in regard to their mothering, it is vital to begin from their own experiences and to locate their experiences within a wider understanding of motherhood as a social institution [10]. Two consistent themes within the women’s narratives were that of a simultaneous increased sense of responsibility with regards to their children (including but not limited to the protection of their children), as well as a loss of control over their mothering, as mirrored in that of [15] study with 26 abused mothers in the United Kingdom. Within our research, the women explained that aspects of their mothering role were out of their control due to the controlling behavior of their partners. Male coercive control is well described in the literature as a central feature of IPV, which includes intimidation, humiliation, degrading and multiple other tactics [26], as demonstrated in this study. Prior studies have also shown the use of children by perpetrators to exert power within a relationship in order to undermine the woman’s role as a mother [15, 32]. This pattern of control is in line with research exploring the fathering behaviours of perpetrators, which has shown that they tend to be authoritarian, while also neglectful, and uninvolved regarding their children [17, 33]. Indeed, this research mirrors research conducted in the Asian context [34], which has shown that it is not uncommon for abusive men to assert rights and privileges, such as being the sole rule maker, without accompanying reciprocal responsibilities [16, 27]. For example, the women in this study explained that their partner’s lack of assistance extended to parenting duties and therefore, mothers found themselves having no option but to parent with minimal support, consistent within previous research [18]. Overall, our findings support the notion that men’s attacks on a women’s’ mothering and mother–child relationships are fundamental in their quest for domination and control. The loss of control over their mothering which at the same time increased their sense of responsibility for their children created a conflict for women. This potentially creates a tense context whereby mothers expect themselves to meet certain motherhood expectations, while having less control over means and resources (including financial), which may lead to a basket of adverse emotions [14].

Our research shows that mothers experiencing IPV may assess themselves negatively through normative paradigms of ‘good mothering’, despite that they often mother in the best way they know how to, given challenging circumstances. This is important as abusive men may exploit women’s desires to be good mothers [27]. For example, the mothers in this study experienced feelings of failure, guilt, and inadequacy in not being able to protect their children. This is consistent with previous South African research that has found that experiences of IPV can undermine a woman’s confidence in her ability to adequately care for and protect her children from harm [35]. The societal notions of the ‘good mother’ which were founded upon idealized versions of motherhood may especially create additional tension for women who mother within the context of IPV [27]. This is because this narrative sets an expectation for women to self-sacrifice and provide unwavering care and protection. However, women in abusive relationships may themselves need protection, care, and support to mother [36]. Thus, women who mother within such circumstances, are especially vulnerable to adverse mental health [35, 36].

Another compelling theme within the mothers’ narratives, was that of a ‘double level of intentionality’, which refers to an act aimed at either the mother or the child, which is simultaneously meant to affect the other [37]. This study illustrated that the mothers endured emotional trauma via witnessing their children being abused, while simultaneously needing to remain silent, and being unable to protect their children when they were abused, all of which may have been intended to harm the mothers emotionally and psychologically., Men may hurt children in order to hurt the mother [27] and recent South African research has found that abusive men may even go as far as to kill children in an attempt to harm their female partners [38]. It has been explained that such perpetrators are aware that harming the child, will cause intense and long-term emotional harm to their spouse, and so act in a way consistent with these mental states [38]. A large body of work has explored the overlapping occurrence of IPV and child maltreatment mainly from the global North [39]. However more recently studies on the recognition of the intersection of child abuse and IPV have emerged from developed settings. Research from Asian and African countries, such as, India [40], Iraq [41], the Philippines [42], Thailand [43], Vietnam [44], and Uganda [45], have documented the co-occurrence of child abuse and IPV.

Research with perpetrators/fathers [24] revealed that they tend to purposefully attack mother–child relationships to be ‘hurtful’ and ‘abusive deliberately. You may ask, ‘why her mothering? It was just to assert power over her, attacking something that probably means the most to her’ [27, pp. 8–9]. Thus, we are reminded that, ‘It is not an accident that abusive men attack women’s abilities to mother, they know that this represents a source of positive identity, the thing above all else that abused women try to preserve, and also that it is an area of vulnerability’ [24, pp.158].

This study has shown that part of supporting abused women is also understanding the impact of being a mother in a violent relationship. Support for abused women, should therefore include provisions of skills and assistance to understand how the violence they endured, worked to disrupt their role as mothers. Care should also be given so that support staff, family, and friends do not blame or shame women for their maternal behaviour while in the abusive relationship and should strive to reduce this pressure. Thus, interventions should focus on equipping mothers with tools, knowledge, and resources, which could potentially assist them, for example, to regain control over their mothering, thereby, attempting to increase positive emotions, and feelings of empowerment, parental adequacy, and overall confidence. Health workers are encouraged to build on the notion of agency and remind the women that even though they may have lost a sense of control while being in the relationship, they ultimately displayed strength, courage, and agency in leaving (if this is applicable). In order to empower abused women and to assist them to gain more control over their mothering, social workers are encouraged to emphasize women’s strengths and the strategies they have utilized in order to protect and care for their children [15]. Overall, this study emphasizes the importance of maternal health interventions, which address mental and physical health issues, related to the abuse, as a means of improving maternal and infant health outcomes [46].

Study limitations include conducting telephonic interviews which may have interfered with the building of rapport with the participants. Another limitation of this study is that we were not able to involve participants in the later stages of the study to verify that the interpretation of the data was sufficiently reflecting their experiences.

This study has expanded on the emerging literature on domestically violent men’s assaults on women as mothers, from the perspective of the women who mothered while being in abusive relationships. Deepening our understanding of this often hidden and not well understood tactic will serve to improve our overall understanding of IPV as it is a shift away from a narrow conceptualization of IPV. Instead, this form of abuse, which encompasses varied tactics, largely aimed at controlling women’s mothering experiences, identities, and practices before and during pregnancy, childbirth, and thereafter, should exist alongside more well-described forms of IPV, such as physical and sexual abuse. This is important so as to avoid practices that are founded upon an arguably incomplete understanding of IPV. To date, the area of mothering while in an abusive relationship, is an area that remains marginalized in the mainstream IPV literature [47]. Interestingly, this form of IPV does not have an established name, is not well theorized, and there is currently an absence of tools with which to measure it and its potential for disrupting the mother-child relationship [27]. Future research is urgently needed, especially within undeveloped settings to assist in developing primary and secondary prevention interventions for both partner and child abuse.

## Supporting information

S1 File Participant quotes. (PDF)
