# Supplementary material for: ‘I’m not the mother I wanted to be’: Understanding the increased responsibility, decreased control, and double level of intentionality, experienced by abused mothers
Source: PLoS One. 2023 Jun 29;18(6):e0287749. doi: 10.1371/journal.pone.0287749 (PMC10309974; doi:10.1371/journal.pone.0287749)
Supplement: S1 File — (PDF) [file pone.0287749.s001.pdf]

Ingrid:

"*While I was pregnant, which was in winter hey, like 8 months pregnant, he* (boyfriend) *would beat me and then make me take my clothes off and kick me out, because he knew I have nowhere to go, then I sleep on the mat where you wipe your feet before coming in*".

"*He refused to give the child a name. I was very disappointed because I thought as a father, he would want to give his son a name. To me it shows that he didn't have any interest".*

"Once my baby was born, he made all the rules, but he did nothing for my baby".

"You know nothing" about being a mother. She said, "*It hurts so much and made me lose confidence in being a mother. It also made me frustrated because even though he was telling me all the time that I don't know what I'm doing, he also wouldn't offer advice or help, but he made rules for the baby*".

Caroline:

"One day he (husband) *threw petrol over me and stood over me and there was a lighter and he was telling me he is going to burn me to death. He kept sitting there and flipping and striking the lighter on and off the whole time. I just froze and I couldn't move because I was so scared".*

"*was like me. They started crying a lot and not talking … just keep everything inside*".

Angel:

"*He* (husband) *would never take no for an answer".*

"*I have nerve damage on my left side so any form of touch on that side is very painful. He liked the fact that I have this disability … because I couldn't defend myself. When I would be asleep, he would take my clothes off and have sexual intercourse with me, and he would always make sure that he is touching my side where he knows the pain is severe*".

"*the sexual abuse would happen right in front of her* (daughter). *As a mother it broke me; that she had to witness that abuse and it was very traumatizing for her*".

"*I would be too afraid to sleep because when I sleep, he forces himself on me*. *I ended up in hospital many times because I was so stressed, so my blood pressure escalated, and I'd have blackouts*".

"*protective mode, trying to always protect".*

Sally:

"*I was employed but … I was actually forced to resign by him* (husband). *He didn't want me to go back because he thought that there is a man that I was sleeping with. I wasn't. He was so paranoid. He sat down with me and forced me to email my boss that I was resigning, and I really loved my job because I could buy the children stuff and I had some independence*".

"*There was often times where it wasn't necessary to hit them and, in these times, he would be smiling, and he told me that he knows it breaks my heart when he hits the children. Like pure evil hey? I know he was doing it to get to me and it worked*".

"agree to keep the peace, but not really agreeing deep down inside".

"*It almost like depends on me, like if I want him to be nice to my children, then I must like be very nice to him. I must make sure he's happy, like have sex with him and make sure his mouth is moving* (provide food), *so that his relationship with them is okay. So, their relationship is like dependent on how I behave towards him: if he is angry with me, the children will bear the effect. If he is mad at me, he will eat the last packet of chips of theirs, he is not bothered, he won't care. He has that I don't care attitude, no feelings. He will shout at them and beat them for no reason. But tomorrow, if I make sure his tummy is full, then he will play with the children*".

"knew what he (husband) *was capable of*".

"him murdering my children".

"blaming the drugs".

"made me so sad, made me feel like I failed…. and felt guilty like I was never a good enough mother because if I was, they would never do that copycat stuff".

Michelle:

"*He* (husband) *accused me of flashing my male guests my boobs … So, it was decided by him that I wasn't going to work no more, and I was forced to resign. I used to be able to take the children out but after that I couldn't afford to*".

"*incredibly difficult*".

"*I'm not the mother I wanted to be because I was not able to protect my children from being abused or from seeing me be abused. I think I will always sit with that guilt and feeling like I was never good enough. I was always failing because we were both always abused*".

Unity:

"*When I saw how he was beating the children, the things that I feel and want to say, I can't even explain. Like if I see him beating them with the belt, it feels like he is beating my body. One of the hardest parts was keeping my mouth shut".*

"He would beat the children for two reasons. One, because he knows it is going to pain me. Two, because he hopes I intervene, and if I do, then he can beat me. So, he would beat them because he knows my weakness, my children, so beating them for unnecessary things just for me to intervene then he can start with me because he never had a real reason of fighting with me because I always did my best to be a good mother and partner".

"When he is about to beat the kids, that's when I feel at my lowest, because I cannot protect them, I cannot even protect myself. When he is beating the kids… I must run because I am next. Then he knows he got what he wanted because he knows I am in pain when he is beating my children.".

"*good enough*".

"did everything he asked".

Anna:

"My daughter told her father: "what you are doing to my mother, you are also doing to me". That was shocking to me. She meant like the pain I am feeling, she is also feeling, like emotionally, you know?".

"*My daughter* (aged 14) *became a lot like me. She was withdrawn and kept to herself*".

Megan:

"*He* (boyfriend) *always used to abuse me in front of them. He used to like, like it if they cried. Then he would feel like his job is done. He has now abused me and made them cry, you see*?".

"*He* (boyfriend) *would teach them* (children) *if someone hits them, they must hit back. I would tell them not to do that. The worst part is that the children would listen to him, and I am not seen. Like what the father says is right and I am wrong. It is very frustrating, I felt like a nobody, like I had no control over how our children was raised*".

"*If my children were naughty, he would say he is the only one that can punish them, not me. According to him, I am a nothing, he doesn't even see me as their mother. But I must bath them, feed them, clothe them. All of that*".

"*Both my sons* (aged 6 and 5) *use to hit their sister* (aged 2) *terribly. Copying their father…. They see what their father is doing to me, so they feel they can hit their sister. It made me feel like I failed as a mother*".

"*What is concerning to me is that if my son at 6 years old has adopted that aggressive behavior, one day he must also take a wife and raise children. I am concerned that it may have an effect on him when he is older, and right now he is already fighting with other boys and even hitting girls*".

Belinda:

"*Sometimes if my daughters upset him, he would take it out on me… The one time they did something to upset him … and I remember him coming down the passage like flying towards me and shouting this is your fault, now your mommy must suffer". Then he hit me in front of the children. He wanted them to see. The children were screaming for him to stop. He told them, "Next time you'll listen hey?" so he actually hit me to make them upset*".

"*Everything was his rules for the kids, but I was the one that had to enforce them and ensure the kids respect it, otherwise he will kill me*".

"raping my daughter".

"blaming the drugs".

"I didn't conversate with my kids. I avoided my children because there is no way that I was going to open up myself to really hear what my child is feeling. I couldn't bear questions or anything from my kids. It was too emotionally difficult. Yoh it made me feel like I wasn't good enough".

Tony:

"He would say, "this is not my baby, you came here pregnant".

"I had so much responsibility on my shoulders hey, but that man gave me no confidence to do anything. He just broke me every day. I always felt like I wasn't good enough as a girlfriend or a mother".

Ursula:

"loneliness"

"he wasn't there for me. He would never accompany me to the clinic for check-ups. Even when I gave birth, he didn't come to visit or bring nappies or clothes for the baby. Then once we got home it continued, he never even held the baby. He helped with nothing".

Marsha:

"He would even go as far as eating the children's last porridge, then there was nothing for them, then I had to go out and beg for anything for my children to eat. Because I knew before I left in the morning, there would be food for them, but when I got back, he has eaten everything, then the children are hungry when I get home. Then the stress and responsibility to find something for them to eat falls on me".

Lauren:

"I had so much on my plate, it was overflowing. I had to try for me and my children not to be abused, I had to tend to my children, like do everything for them, I had to cook and clean, make sure he is happy, and I must think what I can and can't say all the time".

"We got into an argument and then my daughter (age 7 at the time)*, stepped in between us, she said she won't allow him to hit me, and then he pushed her away and she fell to the ground, and he wanted to come for me, and then she got up and took a knife and stabbed him"*.

"When he (aged 6) *asks me something and I don't respond then he will raise his voice. He will make a little fist. Then I would say "no, don't go on like that". He also always wants to hit his sister* (aged 9). *With children it's like "monkey see monkey do". I'm afraid that my daughter will end up with a guy like that. Or my son will be that guy"*.

Elizabeth:

"He would make all the rules for the children, and I hated it. I told him, "you have no interest in the children, you give them no attention, you have no part in their lives, you not even giving them any love, so how can you make decisions for their lives, because you not there for them?"".

"I woke up with my husband lying next to me and he wanted to have sex with me and I refused so he got up and took out a knife. At that moment my son woke up and told his father, "Tonight I will murder you, tonight I am going to commit a murder". I felt like somebody was protecting me".

"*was becoming very abusive towards the other children*".

Lana:

"*My daughter* (aged 5) *would cry a lot and scream when she sees him abuse me. Then next day maybe she would ask me questions like, "why did he do that?". Then, I don't know how to answer, like I don't even know how to explain it to her, like I don't have the energy. That also made things worse and reminded me how I failed as a mother*".
